# Tocotrienol-rich fraction reduces retinal inflammation and angiogenesis in rats with streptozotocin-induced diabetes

**DOI:** 10.1186/s12906-023-04005-9

**Published:** 2023-06-02

**Authors:** Muhammad Zulfiqah Sadikan, Nurul Alimah Abdul Nasir, Nor Salmah Bakar, Igor Iezhitsa, Renu Agarwal

**Affiliations:** 1 Department of Pharmacology, Faculty of Medicine, Manipal University College Malaysia (MUCM), Bukit Baru, 75150 Melaka, Malaysia; 2 Centre for Neuroscience Research (NeuRon), Faculty of Medicine, Universiti Teknologi MARA, Sungai Buloh Campus, 47000 Sungai Buloh, Selangor Malaysia; 3 Department of Pathology, Faculty of Medicine, Universiti Teknologi MARA, Sungai Buloh Campus, 47000 Sungai Buloh, Selangor Malaysia; 4 School of Medicine, International Medical University, Bukit Jalil, 57000 Kuala Lumpur, Malaysia; 5 Department of Pharmacology and Bioinformatics, Volgograd State Medical University, Pavshikh Bortsov sq. 1, Volgograd, 400131 Russia

**Keywords:** Tocotrienol-rich fraction, Diabetic retinopathy, Inflammation, Neurodegeneration, Angiogenesis

## Abstract

**Background:**

Diabetic retinopathy (DR) is the second commonest microvascular complication of diabetes mellitus. It is characterized by chronic inflammation and angiogenesis. Palm oil-derived tocotrienol-rich fraction (TRF), a substance with anti-inflammatory and anti-angiogenic properties, may provide protection against DR development. Therefore, in this study, we investigated the effect of TRF on retinal vascular and morphological changes in diabetic rats. The effects of TRF on the retinal expression of inflammatory and angiogenic markers were also studied in the streptozotocin (STZ)-induced diabetic rats.

**Methods:**

Male *Sprague Dawley* rats weighing 200–250 g were grouped into normal rats (N) and diabetic rats. Diabetes was induced by intraperitoneal injection of streptozotocin (55 mg/kg body weight) whereas N similarly received citrate buffer. STZ-injected rats with blood glucose of more than 20 mmol/L were considered diabetic and were divided into vehicle-treated (DV) and TRF-treated (DT) groups. N and DV received vehicle, whereas DT received TRF (100 mg/kg body weight) via oral gavage once daily for 12 weeks. Fundus images were captured at week 0 (baseline), week 6 and week 12 post-STZ induction to estimate vascular diameters. At the end of experimental period, rats were euthanized, and retinal tissues were collected for morphometric analysis and measurement of NFκB, phospho-NFκB (Ser536), HIF-1α using immunohistochemistry (IHC) and enzyme-linked immunosorbent assay (ELISA). Retinal inflammatory and angiogenic cytokines expression were measured by ELISA and real-time quantitative PCR.

**Results:**

TRF preserved the retinal layer thickness (GCL, IPL, INL and OR; *p* < 0.05) and retinal venous diameter (*p* < 0.001). TRF also lowered the retinal NFκB activation (*p* < 0.05) as well as expressions of IL-1β, IL-6, TNF-α, IFN-γ, iNOS and MCP-1 (*p* < 0.05) compared to vehicle-treated diabetic rats. Moreover, TRF also reduced retinal expression of VEGF (*p* < 0.001), IGF-1 (*p* < 0.001) and HIF-1α (*p* < 0.05) compared to vehicle-treated rats with diabetes.

**Conclusion:**

Oral TRF provided protection against retinal inflammation and angiogenesis in rats with STZ-induced diabetes by suppressing the expression of the markers of retinal inflammation and angiogenesis.

## Background

Diabetic retinopathy (DR) is a common microvascular complication of diabetes that affects one-third of diabetic population and is the leading cause of blindness in people over the age of 50 [1, 2]. About 35 to 60% of DR patients progress to the advanced stage of the disease, proliferative diabetic retinopathy (PDR), which involves neovascularization, vitreous hemorrhage, proliferative membrane formation, iris neovascularization, and may even lead to glaucoma. Patients can, in fact, lose their vision within 10 years of diagnosis [3, 4].

Inflammation is an important factor in the development of PDR [5, 7]. The activity of inflammatory cytokines such as interleukin-1 beta (IL-1β) and tumor necrotic factor-alpha (TNF-α) as well as the pro-inflammatory transcription factor, nuclear factor kappa B (NFκB), has been found to be significantly increased in diabetic retina [8]. NFκB, a heterodimer with a subunit p65, modulates transcription of several genes that are involved in immune response and inflammation [9, 12]. Suppression of NFκB activation reduces pro-inflammatory cytokines expression, including interleukin-6 (IL-6). High level of IL-6 is associated with breakdown of blood retinal barrier (BRB) and retinal vascular changes in hyperglycemic environment [13].

The processes of inflammation and angiogenesis are interconnected in progressive DR. Higher inflammatory cytokines expression promotes expression of vascular endothelial growth factor (VEGF), an angiogenic marker [14]. On the other hand, VEGF induces expressions of inflammatory cytokines such as TNF-α, IL-1β and IL-6 [15]. VEGF is the most recognized angiogenic marker in DR and its expression increases with increase in the severity of the disease [16]. Current medical treatment of DR achieves a reduction in VEGF signaling by using monoclonal antibody that block the binding of VEGF to its receptors. Anti-VEGF treatment has been shown to successfully slow down the PDR development [17]. The expression of VEGF increases under hypoxic conditions due to increased activity of hypoxia-inducing factor (HIF)-1, a transcription factor and essential oxygen sensor in tissues [18, 19]. In hypoxic tissue, the degradation of HIF-1α is inhibited, therefore, its expression is elevated [20, 21]. Higher expression of HIF-1α in the vitreous humor of PDR patients compared to those without DR or NPDR patients has been reported [22]. Lower HIF-1α expression is associated with reduced VEGF expression [23].

Insulin-like growth factor (IGF-1) is another modulator of VEGF expression [24]. Transgenic mice overexpressing IGF-1 have higher VEGF expression in the retinal glial cells [25], which supports the notion that IGF-1 expression induces VEGF transcription [26, 27]. Other than promoting angiogenesis, IGF-1 also modulates neuroinflammatory responses [28]. It regulates neuroinflammatory changes through promoting a switch to microglial phenotype [29].

Since inflammation and angiogenesis play a critical role in DR pathogenesis, substances that can target both pathways could be effective in preventing the onset and/or progression of this disease. Tocotrienol-rich fraction (TRF) is a potent antioxidant and consists of 80% tocotrienol and 20% tocopherol. Previous studies have shown its beneficial effects in inflammatory conditions such as atherosclerosis [30], diabetic neuropathy [31], non-alcoholic fatty liver disease [32], gastric mucosal lesions [33], diabetic nephropathy [34], and osteoporosis [35]. TRF was also shown to possess anti-cancer properties due to its anti-angiogenic effects in hepatocellular carcinoma [36], colorectal adenocarcinoma [37], mammary cancer [38], gastric cancer [39] and prostate cancer [40]. Additionally, TRF possesses anti-diabetic property and has been shown to improve glycemic control [41]. Furthermore, it improves renal [42] and vascular functions [43]. Since its effects in suppressing retinal inflammation and angiogenesis remain relatively unexplored, in this study, we studied the effects of TRF against retinal inflammation and angiogenesis in streptozotocin (STZ)-induced diabetic rats.

## Materials and methods

### Animals

The study was approved by Institutional Ethical Committee (Ethical Approval No: UiTM CARE 3/2019/(286/2019)) and all animal handling complied with Associations for Research in Vision and Ophthalmology (ARVO) statement for the use of animals in ophthalmic and vision research. Male Sprague-Dawley rats (200–250 g) were housed on a 12-hour light / dark cycle with access to food and water ad libitum. Rats were acclimatized for a week and underwent systemic and eye examination before commencing the study. Those found normal were included in the study.

### Induction of diabetes

Rats were fasted overnight prior to intraperitoneal (i.p.) injection of STZ (Cat. No. sc-200,719, Santa Cruz Biotechnology Inc., CA, US) for induction of diabetes. For injection, STZ was dissolved in an ice-cold sodium citrate buffer (10 mmol/L, pH 4.5) and was given at a dose of 55 mg/kg body weight [44]. Blood from the tail vein was collected 48 h after injection to estimate blood glucose levels using the Accu Chek Performa glucometer (Roche Diagnostic, Rotkreuz, CH). Rats with a blood glucose level of more than 20 mmol/L were considered as diabetic [45]. Normal rats similarly received i.p. injection of sodium citrate buffer.

### Study design

Animals were divided into three groups that consisted of nondiabetic rats treated with vehicle (N), diabetic rats treated with vehicle (DV), and diabetic rats treated with TRF (DT). A total of 130 rats were included in the study, of which 36 rats were nondiabetic control rats. The rest of the 94 rats were injected with STZ to induce diabetes. Among STZ-injected rats, 6 rats had blood glucose lower than 20 mmol/L and were not included in further study. Rats with blood glucose level of more than 20 mmol/L were considered diabetic and were randomly divided into DV and DT. However, during experimentation, 16 diabetic rats (8 rats from both DV and DT) developed an infection and died. The rest of the 72 rats remained diabetic and survived (*n* = 36 for each diabetic group). TRF was given orally in a dose of 100 mg/kg body weight [46] in the DT, whereas DV and N received olive oil, which was used as a vehicle. The palm oil-derived TRF used in this study, obtained from ExcelVite Sdn Bhd, Perak, MY, contains all isoforms of tocotrienol and α-tocopherol (EVNol™ 50%; 12.3% α-tocopherol, 13.1% α-tocotrienol, 2.1% β-tocotrienol, 19.4% γ-tocotrienol and 5.8% δ-tocotrienol).

Treatment was started 48 h post-STZ injection and was given by oral gavage once daily for a period of 12 weeks. Blood glucose levels and body weight were monitored weekly during the experimental period. Fundus images were captured at baseline (week 0), weeks 6 and 12 post STZ-induction. After 12 weeks of treatment, animals were euthanized with sodium pentobarbital (50 mg/kg, i.p.; Cat. No. 02095-04, Nacalai Tesque, Kyoto, JP) for eyeball enucleation and retinal collection. Eyeballs were preserved for histological examination whereas retinal tissues were preserved for subsequent biochemical analysis. Four eyeballs from 4 different animals were used for hematoxylin and eosin (H&E) (*n* = 4) and immunohistochemical (IHC) staining. Sixteen retinas from 8 different animals (2 retinas were pooled together, *n* = 8) were subjected to multiplex enzyme-linked immunosorbent assay (ELISA) to determine the retinal expression of IL-1β, IL-6, IFN-γ, TNF-α and MCP-1. Similarly total NFκB and phospho-NFκB expression was determined using 16 retinas by ELISA; Another set of 16 retinas was used to determine expression of iNOS, VEGF, IGF-1 and HIF-1α using ELISA. Real-time quantitative polymerase chain reaction was used to determine gene expression of various markers using 16 retinas from each group.

### Fundus imaging

**Fig. 1:**
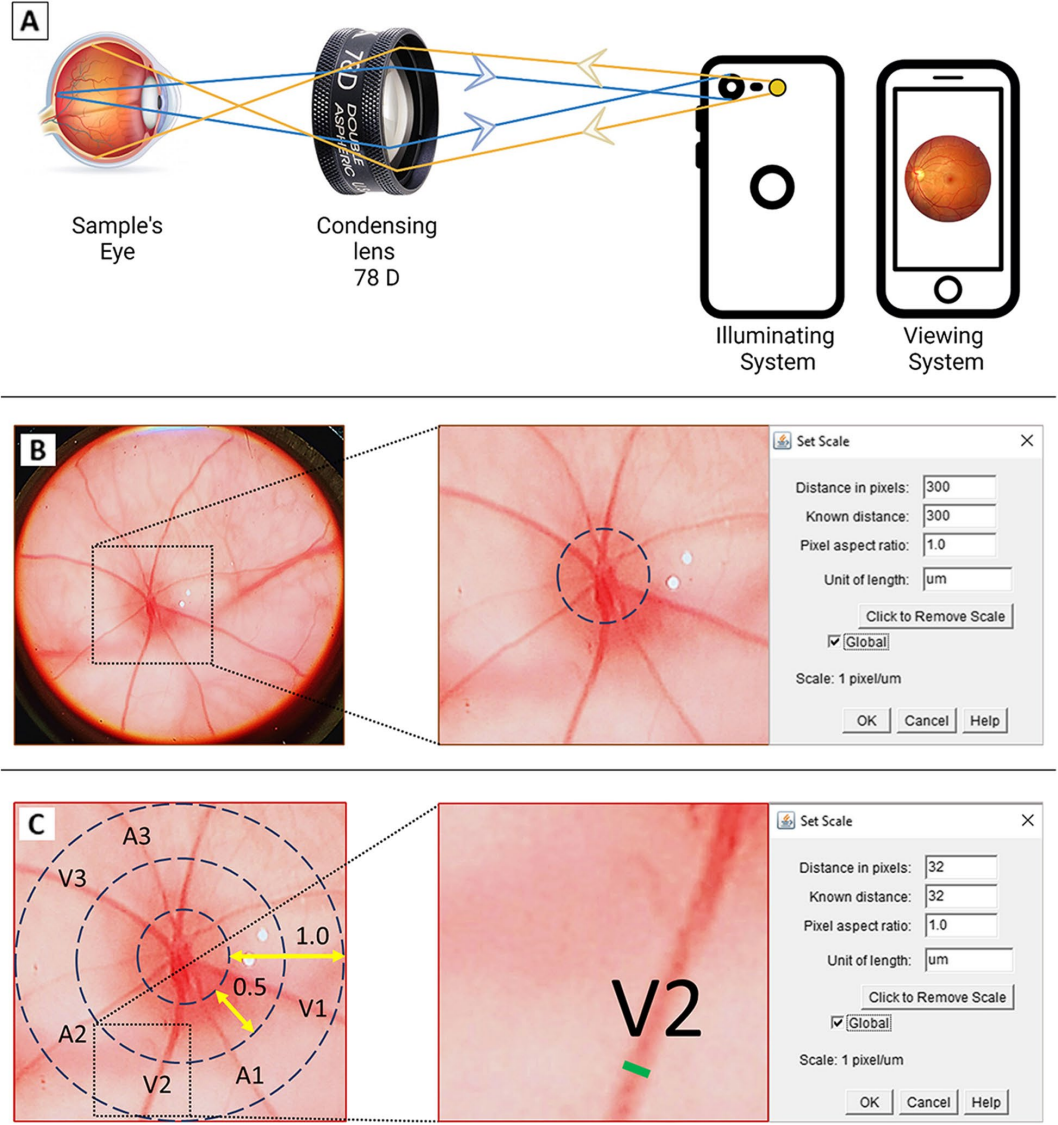
Representative images for the measurement of vessel diameter using ImageJ. **A **Representative image showing the utilization of 78D lens in capturing the fundus image; **B** Image calibration by setting a scale bar with 300× magnifications; **C **The diameter of retinal vessel that crossed through the circumferential zone of 0.5-to-1-disc diameter from the optic disc margin . Blue arrow: light rays forming real inverted fundus image, yellow arrow: illuminating light rays from light source, green line: venous diameter, V: venous, A: arterial

The technique used for fundus imaging was as described previously [47]. Rats were anesthetized with sodium pentobarbital (50 mg/kg, i.p.) and tropicamide (Cat. No. NDC:0998-0355-15, Mydriacyl® Alcon, Geneva, CH) was instilled onto the eye for pupillary dilation 30 min prior to imaging. The optic disc was aligned to the field of view. Fundus was visualized using diopter lens (78D, Volk Optical, Ohio, US) for small animals and images were captured using a smartphone camera (iPhone 7 Plus, Apple Inc., Cupertino, California, US) (Fig. 1A). The lens was positioned 1 cm away from the rat’s eye and the camera was positioned 8 cm away from the lens.

Captured fundus images were set at 3456 × 4184 pixels, 1 μm per pixel [48] in JPEG format, and were transferred into the Fiji ImageJ software (version 2.5.0, NIH, US) [49]. Poor quality images with less than three main retinal vessels (both veins and arteries) observable within the optic disc were excluded. The veins were recognized by their dark red color with broader caliber whereas, the arteries had bright red color with smaller caliber [50]. The images were calibrated by fixing the diameter of the optic disc at 300 μm, which was based on the optic disc average diameter as reported by Cohen et al. [51] (Fig. 1B). The vessel diameter analysis process was adapted from Sadikan et al. [52]. A circumferential zone was constructed at 0.5- and 1-disc diameter from the optical disc margin (Fig. 1C). The diameter of three widest veins and three widest arteries that coursed through the zone between 0.5- and 1-disc diameter were measured in micrometer and their average values were then calculated. The image analysis was done by two independent, blinded researchers.

### Retinal morphometric analysis

The methods of eyeball fixation, sectioning, and staining were as described by Sadikan et al. [53]. The enucleated eyeballs were fixed in 10% neutral buffered formalin for 24 h, and this was followed by paraffin embedding. Tissue sections at a thickness of 3 µM were taken at 1 mm from the temporal edge of the optic disc and were subjected to H&E staining. The stained retinal sections were examined by two independent observers under a light microscope at 20× magnifications (Olympus IX8, Olympus Corporation, Tokyo, JP). Five random areas from each retinal section were selected and captured using imaging software (NIS-Elements Basic Research, version 4.30, Nikon Instrument Inc., Tokyo, JP) as described by Sadikan et al. [54]. The morphometric measurements on the retinal sections were performed using Image J software (Image J 1.31, National Institutes of Health, Bethesda, MD, US). The measurements included: (1) thickness of ganglion cells layer, (2) thickness of the inner plexiform layer (IPL), (3) thickness of the inner nuclear layer (INL), and (4) thickness of outer retina (measured between the inner edges of the outer plexiform layer and inner boundary of the RPE) in micrometer (µm). The average of the measurements by two independent observers was used for analysis.

### Retinal total NFκB, phospho-NFκB and HIF-1α expression using immunohistochemical (IHC) staining

The IHC was performed using 2-step plus poly-HRP anti-rabbit/mouse IgG detection kit (Cat. No E-IR-R213, Elabscience Biotechnology, Houston, Texas, US). After the tissue sections were dewaxed and washed in phosphate-buffered saline (PBS), antigens were retrieved by immersing the slides in the antigen retrieval solution (10 mM sodium citrate, 0.05% Tween 20, pH 6.0) at boiling point for 20 min. Slides were then cooled down to room temperature for 1 h. Slides were rinsed with PBST (1X PBS, 0.1% Tween 20) and incubated with 3% hydrogen peroxide for 15 min at room temperature in a humid chamber to block endogenous peroxidase activity. The slides were then incubated with normal goat serum as blocking buffer for 30 min at 37 °C followed by anti-NFκB antibody (1:100; Cat. No. ab16502, Abcam Biotechnology, Cambridge, UK) in a humid chamber overnight at 4 °C. After washing with PBST, the slides were incubated with polymer helper containing antibody enhancer for 20 min at 37 °C followed by incubation with secondary antibody containing polyperoxidase-anti-mouse/rabbit IgG (Cat. No. E-IR-R213C, Elabscience Biotechnology) for 30 min at 37 °C. Slides were stained with 3,3′-diaminobenzidine tetrahydrochloride for 20 min. The slides were then counterstained with Mayer’s hematoxylin. Similar steps were applied to immunostain phospho-NFΚB (1:50; Cat. No. ab86299, Abcam Biotechnology), and HIF-1α (1:100; Cat. No. E-AB-31,662, Elabscience Biotechnology) with similar incubation time. All slides were independently assessed by two observers according to the criteria described by Wu et al. [55]. The positively stained nuclei in GCL were counted in eight randomly selected fields of view at 20× magnification using Fiji ImageJ software.

### Retinal total NFĸB, phospho-NFĸB, iNOS, VEGF, IGF-1 and HIF-1α level using ELISA

The NFκB and phospho-NFκB protein levels were determined using the NFκB p65 (Total/Phospho) InstantOne™ ELISA kit (Cat. No. 85-86083-11 Thermo Scientific, Waltham, Massachusetts US). Collected retinas were rinsed with ice-cold PBS (0.01 M, pH 7.4) and then homogenized using an ultrasonic homogenizer in RIPA buffer with protease inhibitor in a ratio of 1 mg of retinal weight to 10 µL RIPA buffer. For the total NFκB p65 assay, 50 µL of standard, control and samples were incubated with 50 µL of antibody cocktail containing an equal volume of NFκB p65 (Total) Capture Antibody Reagent and NFκB p65 (Total) Detection Antibody Reagent for 1 h at room temperature on a microplate shaker at 300 rpm. Similar steps were also used for detection of phospho-NFκB p65 (Ser536). Then, 3,3’,5,5’-tetramethylbenzidine (TMB) Substrate was added, and the wells were incubated at room temperature for 30 min on a microplate shaker at 300 rpm. Sulfuric acid (0.16 M) was added to stop the reaction and the absorbance was measured using a Victor X5 microplate reader (Perkin Elmer, Waltham, MA, US) at 450 nm. NFκB p65 and phospho-NFκB Ser536 levels were expressed as relative optical density/mg protein.

The levels of retinal iNOS, VEGF, IGF-1 and HIF-1α protein were measured using the commercially available ELISA kit (Cat. No. E-EL-R0520, E-EL-R2603, E-EL-R3001, E-EL-R0513, Elabscience Biotechnology Co., Ltd, US). Collected retinas were rinsed with ice-cold PBS (0.01 M, pH 7.4) and then homogenized in RIPA buffer with a protease inhibitor in a ratio of 1 mg of retinal weight to 10 µL RIPA buffer. Hundred µL of standard, control and sample supernatant was pipetted into the wells which were pre-coated with iNOS/VEGF/IGF-1/HIF-1α antibody and incubated at 37 °C for 90 min. Biotinylated detection antibody was then added and incubated for 1 h. Well plate was washed for 4 times with wash buffer containing 10 mM phosphate buffer pH 7.4, 150 mM NaCl and 0.05% Tween 20. Next, horseradish peroxidase (HRP) conjugate working solution was added and incubated for 30 min. After five rounds of washing process, TMB substrate was added and well plate was incubated at 37 °C for 20 min. The reaction was stopped by adding 0.16 M sulfuric acid and the absorbance was read at 450 nm using a Victor X5 microplate reader.

### Retinal IL-1β, IL-6, IFN-γ, TNF-α and MCP-1 protein level by using Multiplex Immunoassay

A commercially available microparticle (bead)-based multiplex cytokine ELISA kit (Cat. No. RECYTMAG-65 K, Milliplex®, Merck Millipore, Burlington, Massachusetts, US) was used for simultaneous measurement of several cytokines. In this multiplex assay, the microbead is bound to an antibody, which forms antigen-antigen complex that are then detected by a secondary detector antibody (streptavidin) linked with fluorescent reporter (phycoerythrin (PE)) conjugate.

Collected retinas were rinsed with an ice-cold PBS (0.01 M, pH 7.4) and then homogenized by ultrasonic homogenizer in RIPA buffer with a protease inhibitor in a ratio of 1 mg of retinal weight to 10 µL RIPA buffer. For analysis, 50 µL of standard, control and samples were added to the appropriate wells. Twenty-five µL of cytokine detection beads coated with anti-cytokine antibodies (anti-rat TNFα/IL-1β/IL-6/IFN-γ/MCP-1) were added and incubated on a 300-rpm shaker for 18 h at 4 °C. After washing, 25 µL of detection antibodies were added and incubated with agitation on a 300-rpm shaker for 1 h at room temperature. Subsequently, 25 µL of streptavidin-PE was added and incubated for 30 min. After washing twice, 125 µL of Drive Fluid (a specialized lubricant) was added and resuspended for 5 min on 300 rpm shaker. The mean fluorescence of 200 beads per cytokine was used to determine the mean fluorescence intensity of each well. The Magpix Milliplex® Analyst 5.1 software (Merck Millipore, Billerica, US) was used to convert fluorescence readings to cytokine concentrations using a calibration curve.

### Retinal IL-1β, IL-6, IFN-γ, TNF-α, iNOS, MCP-1, VEGF, and IGF-1 gene expression using Real-Time Quantitative PCR (RT-qPCR)

The extraction and purification of RNA was performed using a commercially available spin-column nucleic acid purification kit (Cat. No. GF-TRD-100, Vivantis Technologies Sdn Bhd, Selangor, MY). Samples with RNA concentration of more than 40 ng/µL were considered suitable for DNA conversion. The cDNA synthesis was performed using OneScript® Plus cDNA Synthesis Kit (Cat. No. G236, Applied Biological Materials Inc., Richmond, British Columbia, CA). One µL of 10 mM dNTP Mix and 1 µL of 10 µM random primers were added to the extracted RNA samples. Nuclease free water was added to the dNTP-primer-RNA mixture to make up a total volume of 14.5 µL. The mixture was incubated at 65 °C for 5 min, followed by incubation on ice for 1 min. Master mix containing 5X RT buffer, RNaseOFF Ribonuclease Inhibitor and OneScript RTase were then added. The mixture was then incubated at 25 °C for 10 min, followed by a second incubation at 42 °C for 15 min. The reaction was stopped by incubating the mixture at 85 °C for 5 min. The cDNA was then stored at -20 °C until further use. The primer pair specificity was verified using the Nucleotide Basic Local Alignment Search Tool (BLASTN) and were supplied by Macrogen (Seoul, KR) (Table 1). Stock concentrations were diluted to 10 µM prior to use. The RT-qPCR was performed according to the manufacturer’s protocol. cDNA templates and all the reaction mixture were prepared on ice at 10 µL volume containing 5 µL of BrightGreen 2X qPCR MasterMix, 0.3 µL of forward/reverse primer (10 µM), template DNA and nuclease-free H_2_O. The cycle threshold (Ct) values were measured using Quantstudio 12 K Real Time System (Life Technologies, Thermo Scientific). The relative fold expression of each target genes was determined using the Livak method [56] after normalization to the housekeeping genes, glyceraldehyde 3-phosphate dehydrogenase (GAPDH) and hypoxanthine-guanine phosphoribosyltransferase (HGPRT).

**Table 1:** Primer sequence for tested genes and housekeeping genes

Primer name	NCBI Reference Number	Gene Symbol	Sequence	Annealing temperature (°C)
Interleukin-1 beta	24,494	IL1B_F IL1B_R	CTCCATGAGCTTTGTACAAGG GGGGTTGACCATGTAGTCGT	53.4
Interleukin-6	24,498	IL6_F IL6_R	AAGAAAGACAAAGCCAGAGTC CACAAACTGATATGCTTAGGC	52.8
Tumor necrosis factor-alpha	24,835	TNFA_F TNFA_R	TCAGCCTCTTCTCATTCCTGC TTGGTGGTTTGCTACGACGTG	56.8
Inducible nitric oxide synthase	24,599	INOS_F INOS_R	CACCACCCTCCTTGTTCAAC CAATCCACAACTCGCTCCAA	56.0
Monocyte Chemoattractant Protein-1	24,770	MCP1_F MCP1_R	CTCAGCCAGATGCAGTTAATGC TTCTCCAGCCGACTCATTGG	54.5
Interferon-gamma	25,712	IFNG_F IFNG_R	TATGGAAGGAAAGAGCCTCC TCTGTGGGTTGTTCACCTCG	58.7
Vascular endothelial growth factor A	83,785	VEGFA_F VEGFA_R	CAGCGACAAGGCAGACTATT GTTGGCACGATTTAAGAGGG	55.0
Insulin-like growth factor-1	24,482	IGF1_F IGF1_R	GTGTCCGCTGCAAGCCTAC CAAGTGTACTTCCTTCTGAGTCTTGG	59.0
Glyceraldehyde 3-phosphate dehydrogenase	24,383	GAPDH_F GAPDH_R	CCATGGAGAAGGCTGGGG CAAAGTTGTCATGGATGACC	58.2
Hypoxanthine-guanine phosphoribosyltransferase	24,465	HPRT_F HPRT_R	GACCGGTTCTGTCATGTCG ACCTGGTTCATCATCACTAATCAC	55.7

### Data analysis

Statistical Package for Social Science (SPSS) software version 20.0 was used for all statistical analysis. Data was normally distributed and was confirmed using the Shapiro-Wilk test. All data were expressed as mean ± SD. One-way ANOVA with post-hoc Bonferroni test was applied for multiple comparison analysis.

## Results

### Effect of TRF on body weight gain and blood glucose level

Diabetic rats generally appeared to be thinner and weaker compared to N rats. There was 18.18% mortality rate among diabetic rats compared to 0% among N.

**Fig. 2:**
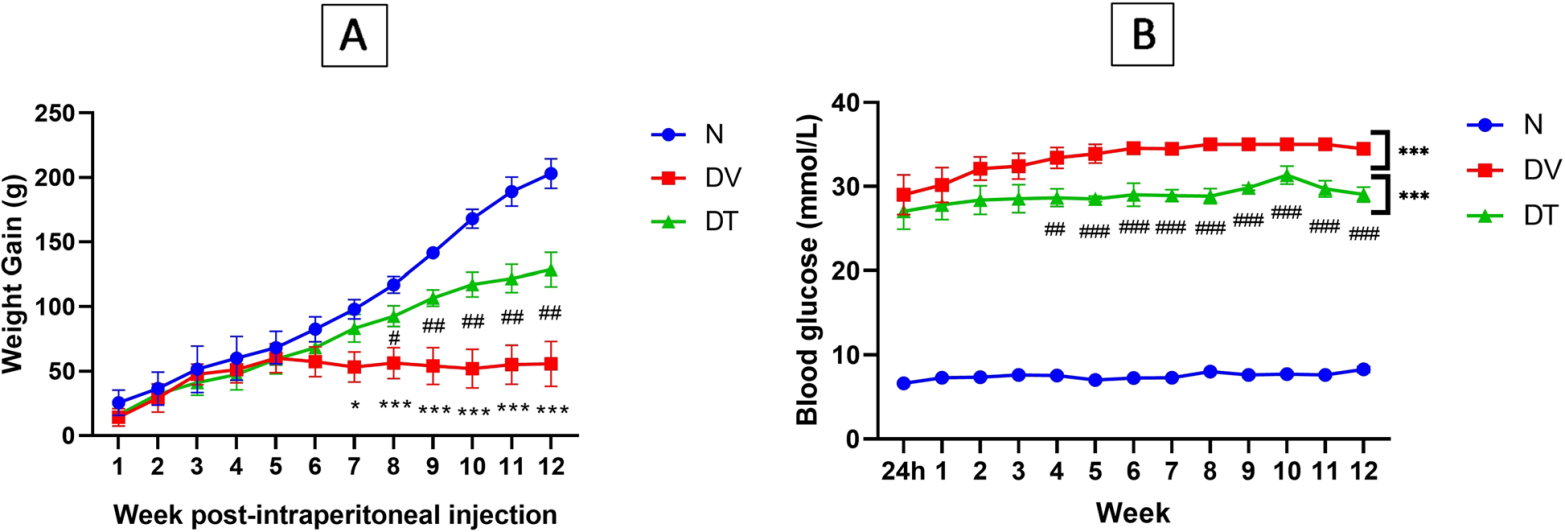
Effect of TRF on (**A**) weight gain (grams), and (**B**) blood glucose level in STZ-induced DR rats. N: Normal rats with vehicle treatment, DV: Diabetic rats with vehicle treatment, DT: Diabetic rats with TRF treatment. Data was parametric and presented as mean ± SD, *p < 0.05/ ***p < 0.05 versus N, ^#^*p* < 0.05 / ^##^*p* < 0.01 / ^###^*p* < 0.001 versus DV. *n* = 8

The weight gain was significantly lower in DV and DT compared to normal control group starting from week 7 to 12 post-STZ-induction. DT showed significantly greater weight gain compared to DV from week 8 post-STZ-induction until end of the experimental period (Fig. 2A). DV showed higher blood glucose level compared to normal control group starting from 48 h post-STZ-induction until the end of experimental period. However, DT exhibited lower blood glucose level compared to diabetic control group starting from week 4 post-STZ-induction until the end of experimental period (Fig. 2B).

### Effect of TRF on retinal layer thickness

**Fig. 3:**
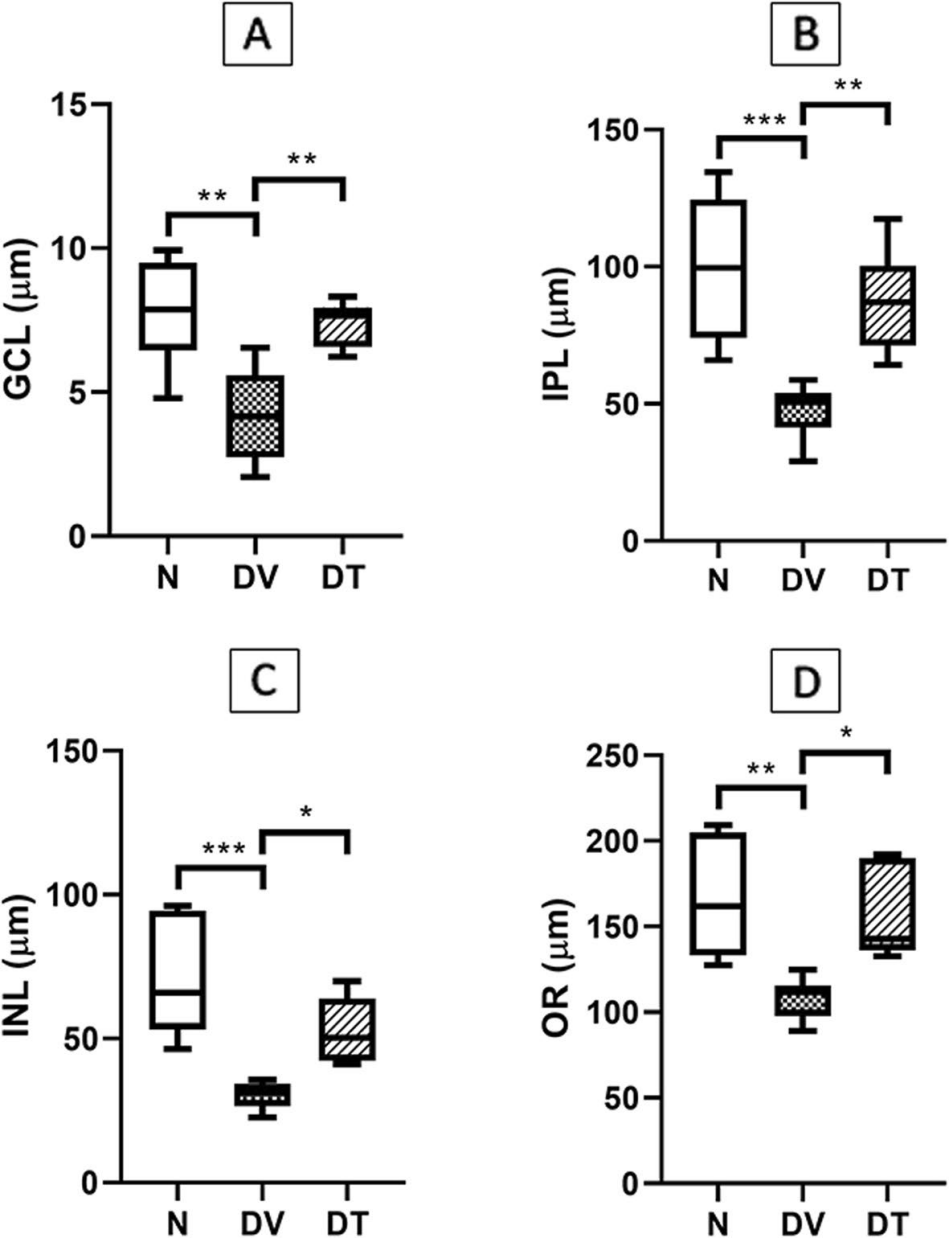
Effect of TRF on the thickness of (**A**) retinal ganglion cells layer (GCL), (**B**) inner plexiform layer (IPL), (**C**) inner nuclear layer (INL), and (**D**) outer retina (OR) in STZ-induced DR rats. N: Normal rats with vehicle treatment, DV: Diabetic rats with vehicle treatment, DT: Diabetic rats with TRF treatment. Data was parametric and presented as mean ± SD, *p < 0.05; **p < 0.01; ***p < 0.001. *n* = 4

The thickness of all retinal layers was significantly reduced in DV compared to N. However, in DT, the thickness of GCL, INL, IPL and OR increased by 1.76-, 1.73-, 1.82- and 1.44-fold, respectively, compared to DV (*p* < 0.01, *p* < 0.01, *p* < 0.05 and *p* < 0.05, respectively) (Fig. 3).

### Effect of TRF on retinal venous and arterial diameter

**Fig. 4:**
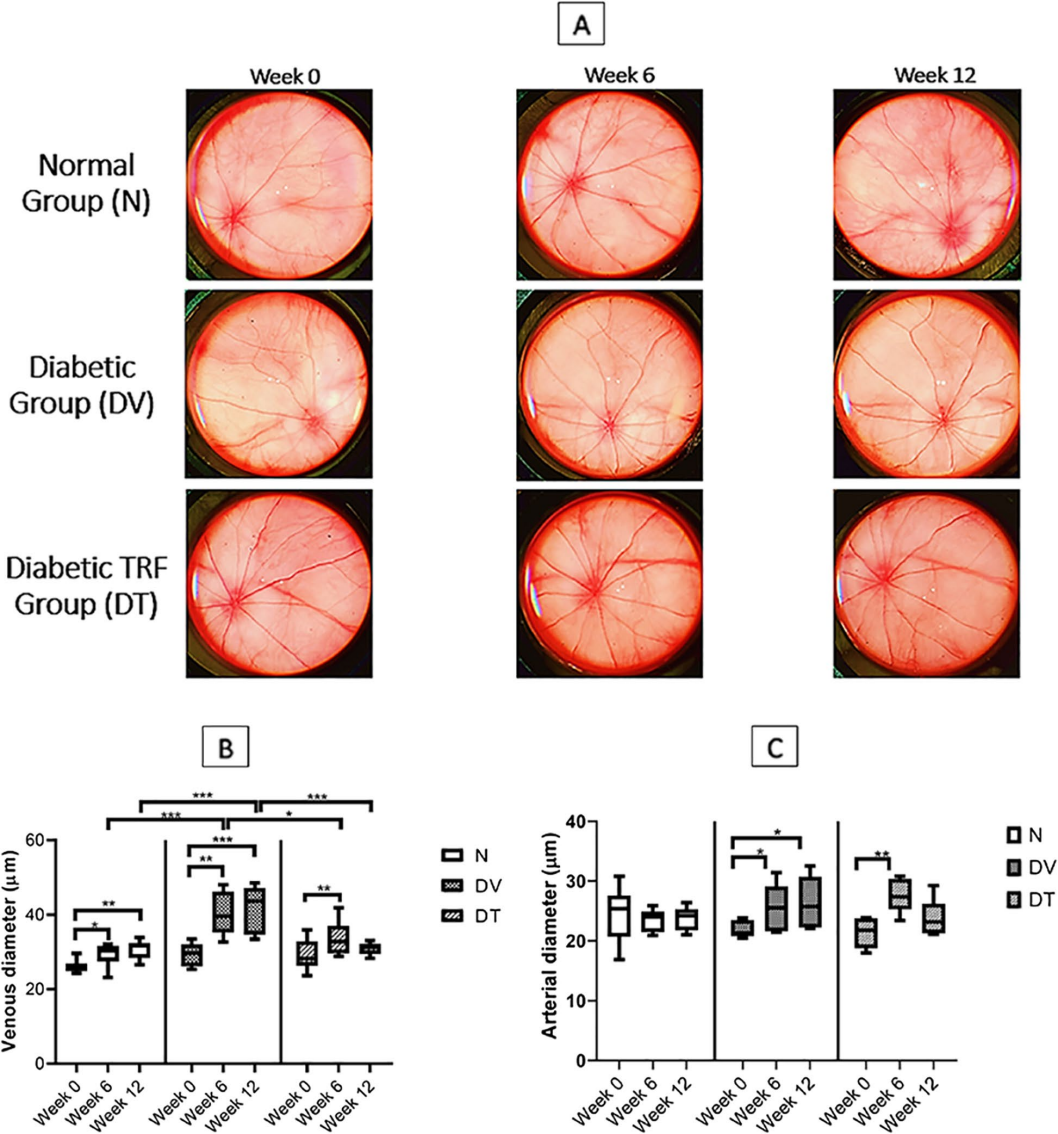
**A** The representative fundus images of rats at week 0, week 6 and week 12 ost-. Effect of TRF on (**B**) retinal venous and (**C**) arterial diameter in STZ-induced DR rats. N: Normal rats with vehicle treatment, DV: Diabetic rats with vehicle treatment, DT: Diabetic rats with TRF treatment. Data was parametric and presented as mean ± SD, *p < 0.05; **p < 0.01; ***p < 0.001. *n* = 8

The representative fundus pictures from all groups are presented in Fig. 4A. At baseline (week 0), the retinal venous diameter among the three groups was comparable to each other. However, it increased in DV and DT at week 6 and 12 compared to the corresponding baseline. Retinal venous diameter remained unchanged from week 6 to week 12 in all groups.

DV demonstrated significantly greater retinal venous diameter compared to that in the N at week 6 and 12 by 1.37-fold (*p* < 0.001) and 1.35-fold (*p* < 0.001), respectively. Retinal venous diameter of DT was smaller than that in the DV at week 6 and week 12 by 1.37-fold (*p* < 0.05) and 1.19-fold (*p* < 0.001), respectively. The difference in the venous diameter at baseline and week 12 in DT was not significant (Fig. 4B).

The retinal arterial diameter among the three groups was comparable to each other at baseline. No significant changes were observed in N throughout experimental period. However, DV showed an increment in the retinal arterial diameter at week 6 and 12 compared to its baseline by 1.17-fold (*p* < 0.05) and 1.20-fold (*p* < 0.05), respectively. DT also showed increment in the retinal arterial diameter at week 6 when compared to its baseline (1.34-fold, *p* < 0.01) but not at week 12. No significant arterial diameter changes were seen among all groups at any time point (Fig. 4C).

### Effect of TRF on the diabetes-induced changes in the expression of markers of retinal inflammation

#### Inflammatory cytokines

**Fig. 5:**
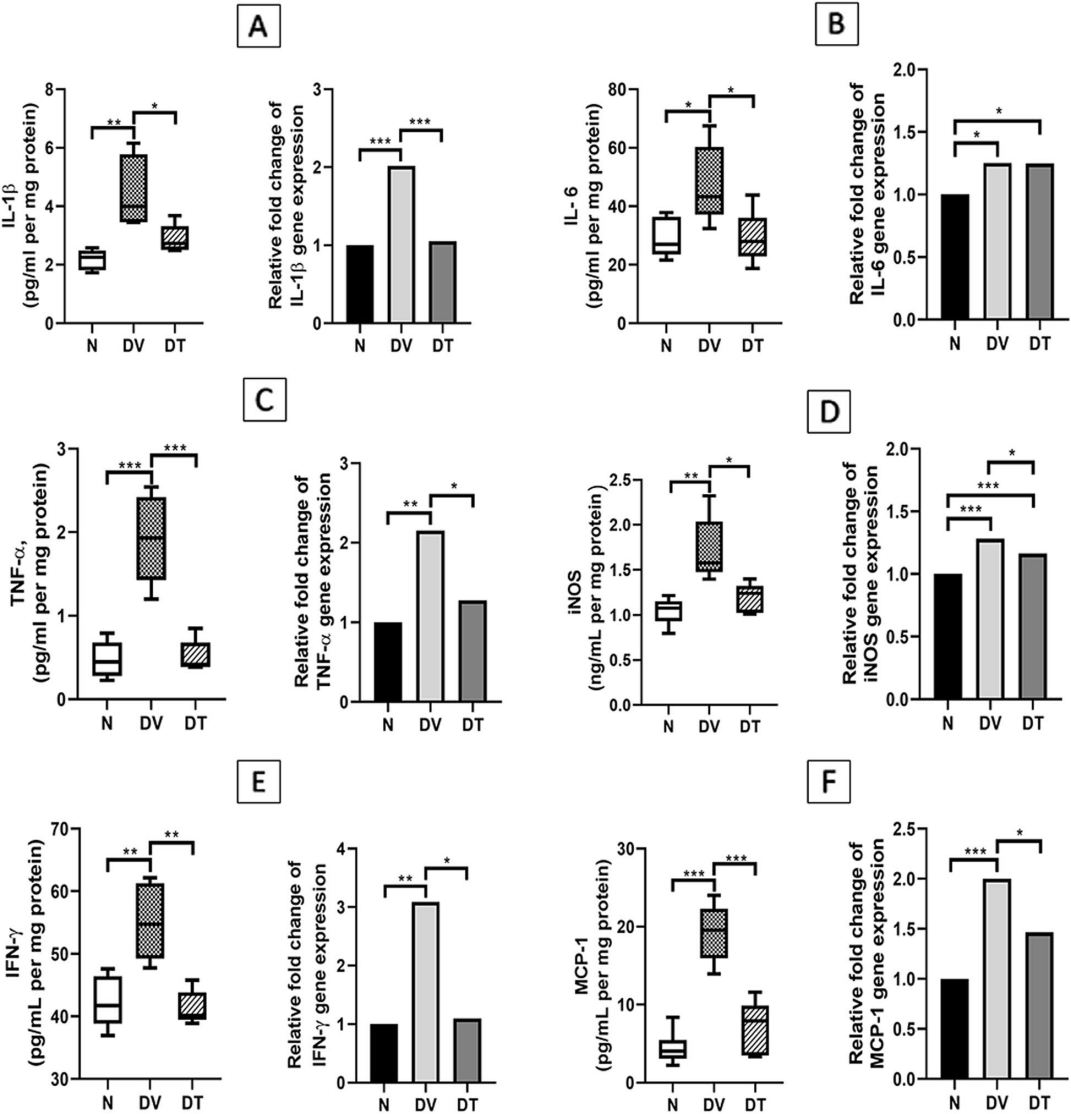
Effect of TRF on the expression of retinal inflammatory markers in STZ-induced DR rats. **A** IL-1β; (**B**) IL-6; (**C)** TNF-α; (**D**) iNOS; (**E**) IFN-γ; (**F**) MCP-1. N: Normal rats with vehicle treatment, DV: Diabetic rats with vehicle treatment, DT: Diabetic rats with TRF treatment. Data was parametric and presented as mean ± SD, *p < 0.05; **p < 0.01; ***p < 0.001. *n* = 8

Retinal IL-1β protein expression in DV was significantly greater compared to N (2.07-fold, *p* < 0.01). However, DT showed 1.56-fold lower IL-1β expression compared to DV (*p* < 0.05). The IL-1β protein expression in DT was comparable to N. IL-1β gene expression as measured by RT-qPCR was significantly higher in DV compared to N (2.02-fold, *p* < 0.001). However, DT showed 1.92-fold lower IL-1β gene expression compared to DV (*p* < 0.001) (Fig. 5A).

Retinal IL-6 protein expression in DV was significantly greater compared to N (1.62-fold, *p* < 0.05). However, DT showed 1.63-fold lower IL-6 expression compared to DV (*p* < 0.05). The IL-6 protein expression in DT was comparable to N. As determined by RT-qPCR, both DV and DT showed greater IL-6 gene expression compared to N (1.25- and 1.24-fold respectively, *p* < 0.05) (Fig. 5B).

Retinal TNF-α protein expression in DV was significantly greater compared to N (4.07-fold, *p* < 0.001). However, DT showed 3.77-fold lower TNF-α protein expression compared to DV (*p* < 0.001). The TNF-α protein expression in DT was comparable to N. Rats in DV showed significantly greater TNF-α gene expression compared to N (2.15-fold, *p* < 0.01). However, DT showed 1.68-fold lower TNF-α gene expression compared to DV (*p* < 0.05). The same in DT was comparable to N (Fig. 5C).

Retinal iNOS protein expression in DV was significantly greater compared to N (1.64-fold, *p* < 0.01). Interestingly, DT showed 1.45-fold lower iNOS protein expression compared to DV (*p* < 0.05), however it was comparable to N. Both DV and DT showed significantly greater retinal iNOS gene expression compared to N (1.28- and 1.16-fold *p* < 0.001). However, DT showed 1.10-fold lower iNOS gene expression compared to DV (*p* < 0.05) (Fig. 5D).

Retinal IFN-γ protein expression in DV was significantly greater compared to N (1.30-fold, *p* < 0.01). However, DT showed 1.33-fold lower IFN-γ protein compared to DV (*p* < 0.01) and this was comparable to N. DV showed significantly greater retinal IFN-γ gene expression compared to N (3.09-fold, *p* < 0.01). However, DT showed 2.82-fold lower IFN-γ gene expression compared to DV (*p* < 0.05) and this was comparable to N (Fig. 5E).

Retinal MCP-1 protein expression in DV was significantly greater compared to N (4.35-fold, *p* < 0.001). However, DT showed 2.78-fold lower MCP-1 protein compared to DV (*p* < 0.001) and this was comparable to N. DV showed significantly greater retinal MCP-1 gene expression compared to N (2.00-fold, *p* < 0.001). However, DT showed 1.36-fold lower MCP-1 gene expression compared to DV (*p* < 0.05) and this was comparable to N (Fig. 5FF).

#### NFκB and phospho-NFκB

**Fig. 6:**
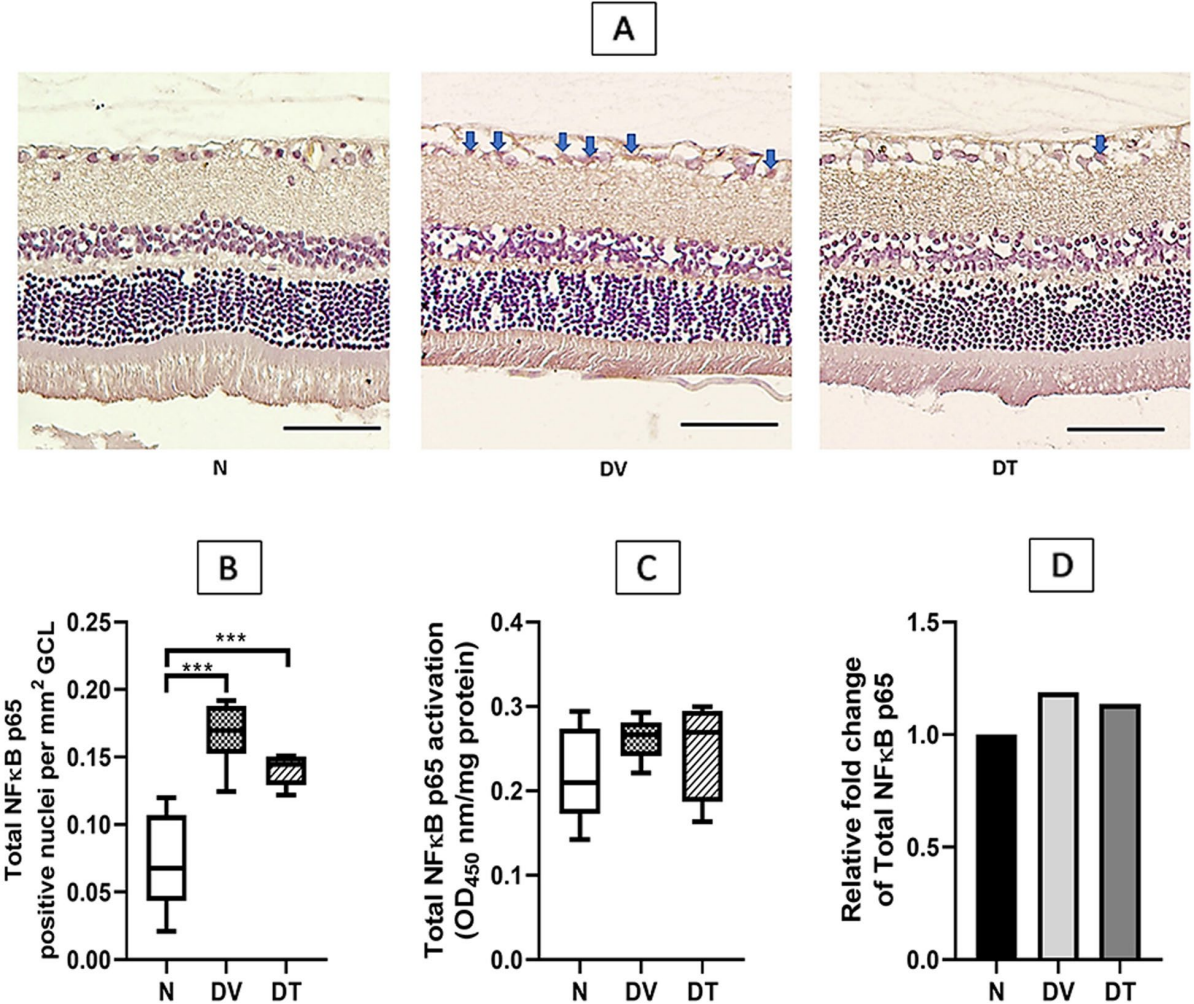
Effect of TRF on retinal NFκB expression in STZ-induced DR rats. **A** Representative immunostained retinal sections showing effect of TRF on retinal NFκB expression; **B** Quantitative expression of retinal NFκB positive nuclei per mm^2^ (*n *= 4); **C **Total NFκB expression measured by ELISA (*n* = 8); **D** Relative fold change of total NFκB. Blue arrows: nuclei positively stained for NFκB. N: Normal rats with vehicle treatment, DV: Diabetic rats with vehicle treatment, DT: Diabetic rats with TRF treatment, GCL: Ganglion cell layer. Data was parametric and presented as mean ± SD, *** *p* < 0.001 (Scale bar: 50 μm) (Magnification 20X)

Representative microphotographs of immunostained retinal section showing expression of NFκB are presented in Fig. 6A. A greater number of NFκB immunostained nuclei were detected in DV compared to N. Quantitatively, the number of NFκB positive nuclei in DV was 2.33-fold higher compared to N (*p* < 0.001). In DT, the number of NFκB positive nuclei remained greater than N (1.96-fold, *p* < 0.001) and it was comparable with DV (1.19-fold, *p* = 0.207) (Fig. 6B).

**Fig. 7:**
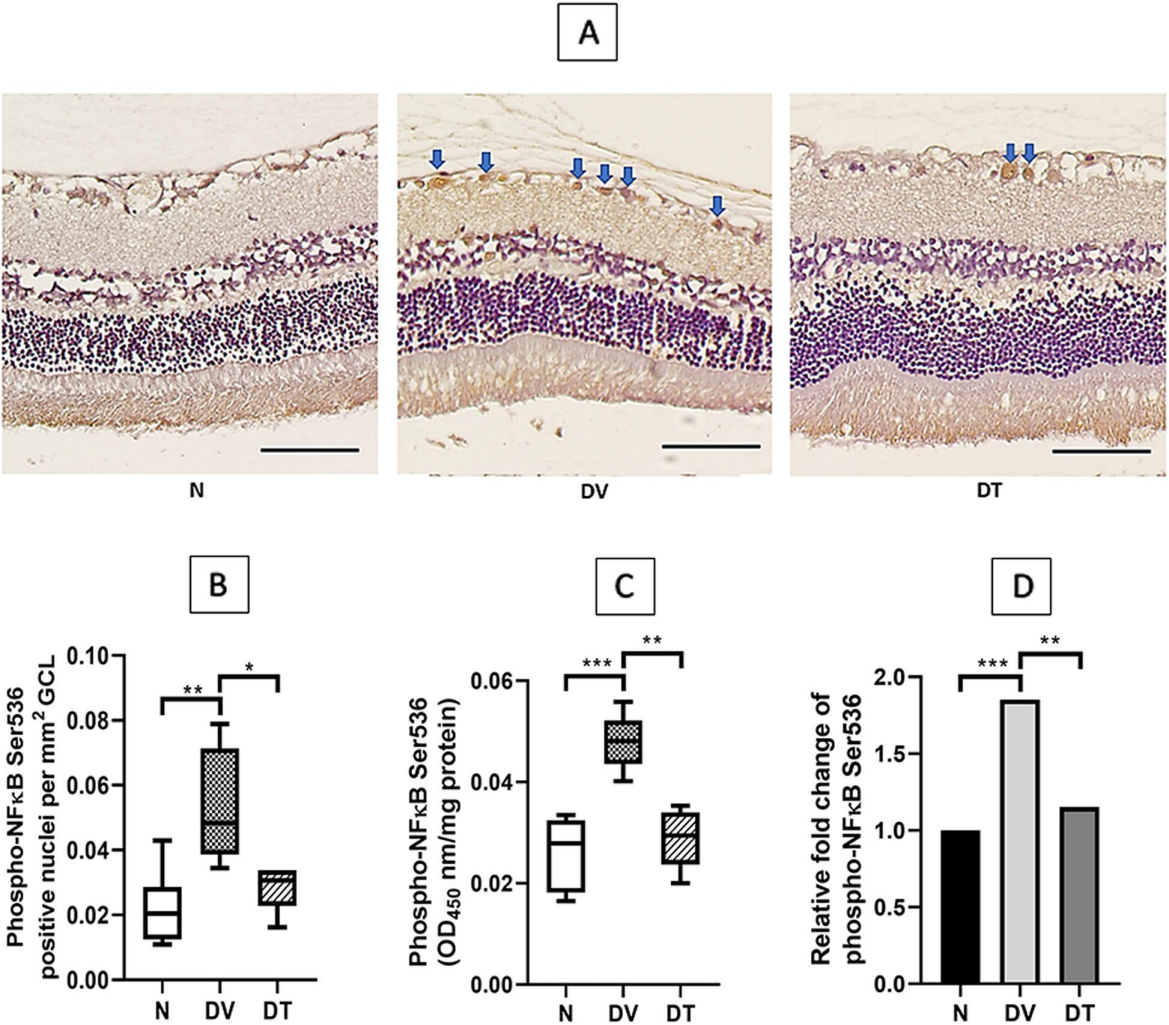
Effect of TRF on retinal phospho-NFκB expression in STZ-induced DR rats. **A** Representative immunostained retinal sections showing effect of TRF on retinal phospho-NFκB expression; **B **Quantitative expression of the effect of TRF on retinal phospho-NFκB positive nuclei per mm^2^ (*n* = 4); **C** Phospho-NFκB expression measured by ELISA (*n* = 8); **D **Relative fold change of phospho-NFκB. Blue arrows: nuclei positively stained for phospho-NFκB. N: Normal rats with oral vehicle treatment, DV: Diabetic rats with oral vehicle treatment, DT: Diabetic rats with oral TRF treatment, GCL: Ganglion cell layer. Data was parametric and presented as mean ± SD, *p < 0.05, **p < 0.01, *** *p* < 0.001 (Scale bar: 50 μm) (Magnification 20X)

Greater immunostaining for phospho-NFκB was observed in DV compared to N (Fig. 7A). Quantitatively, there was 2.42-fold higher phospho-NFκB expression in DV compared to N (*p* < 0.01). Unlike DV, lesser staining for phospho-NFκB was detected in DT and the difference amounted to1.88-fold compared to DV (*p* < 0.05) (Fig. 7B).

Retinal total NFκB protein expression as measured by ELISA did not show any significant differences among the three groups (Fig. 6C and D). Whereas retinal phospho-NFκB expression in DV was significantly greater compared to N (1.85-fold, *p* < 0.001). DT showed 1.65-fold lower phospho-NFκB expression compared to DV (*p* < 0.01) and this was comparable to N (Fig. 7C and D).

**Fig. 8:**
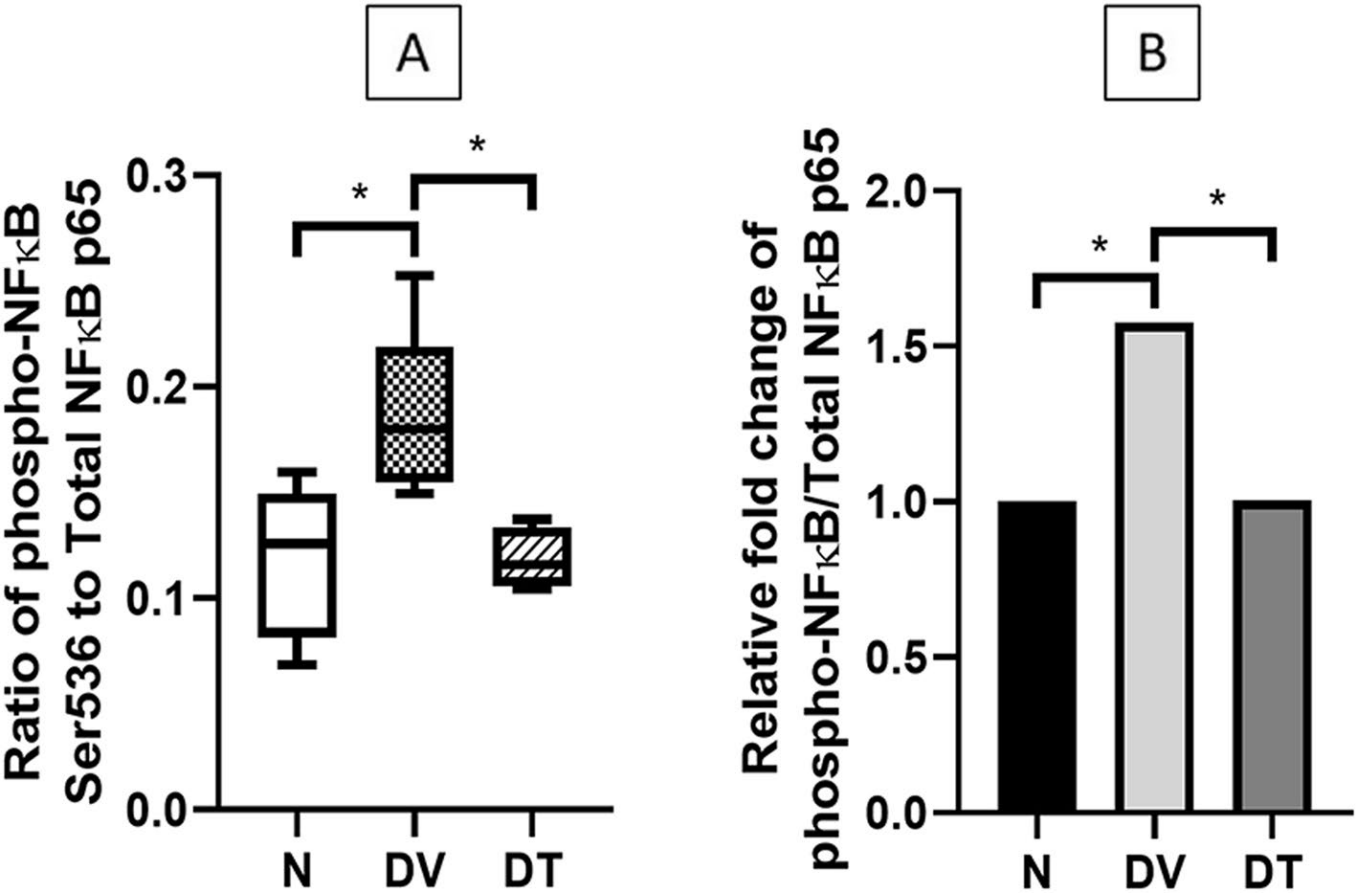
Effect of TRF on the (**A**) ratio of retinal phospho-NFκB to total NFκB in STZ-induced DR rats. **B** Relative fold change of the ratio of phospho-NFκB to total NFκB. N: Normal rats with vehicle treatment, DV: Diabetic rats with vehicle treatment, DT: Diabetic rats with TRF treatment. Data was parametric and presented as mean ± SD, *p < 0.05. *n* = 8

Ratio of retinal phospho-NFκB p65 to total NFκB p65 in DV was significantly greater compared to N (1.58-fold, *p* < 0.05). However, DT showed 1.57-fold lower ratio compared to DV (*p* < 0.05) and this was comparable to N (Fig. 8).

### Effect of TRF on the diabetes-induced changes in the expression of markers of retinal angiogenesis

#### VEGF and IGF-1 expression

**Fig. 9:**
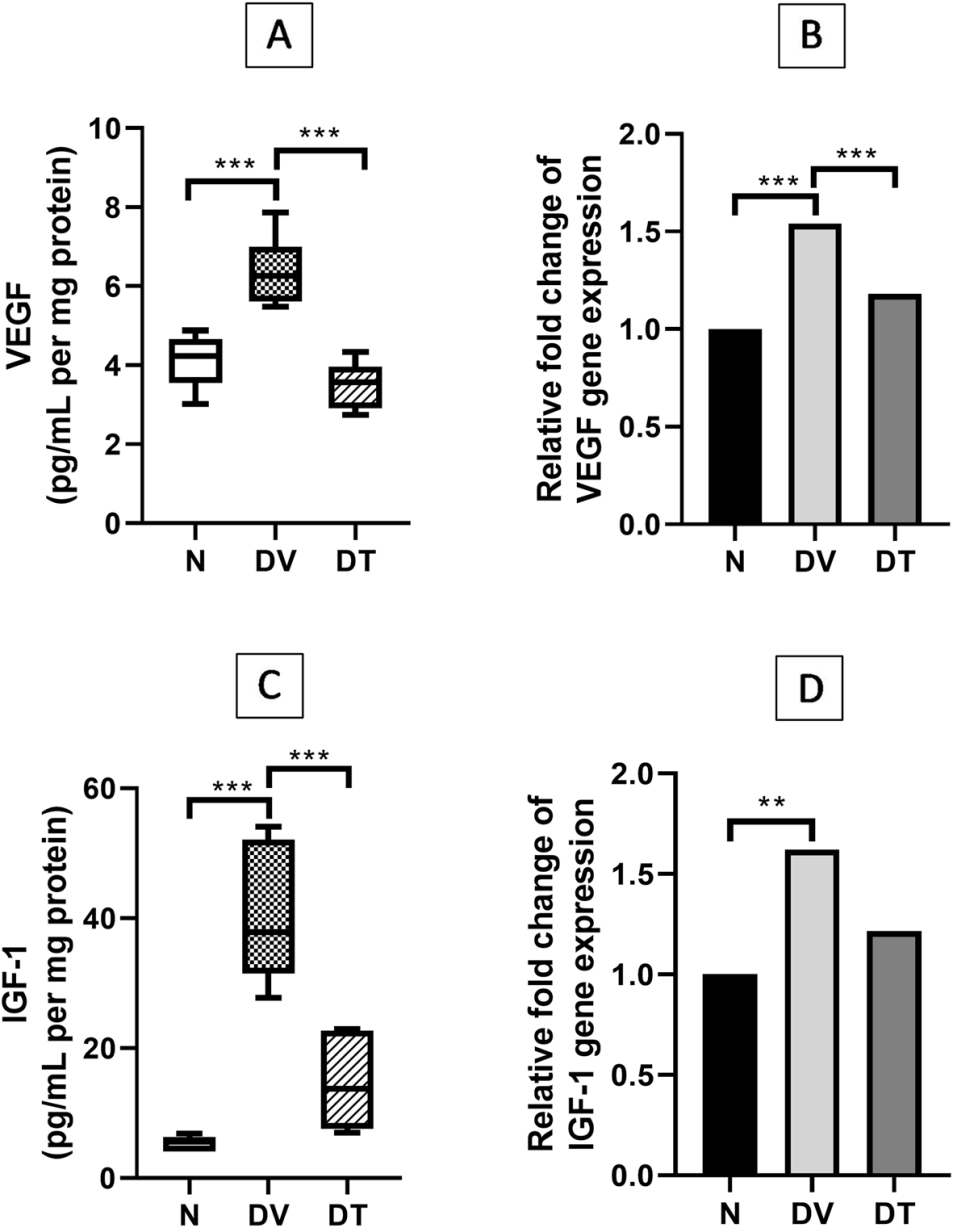
Effect of TRF on retinal (**A**) VEGF protein expression, (**B**) VEGF gene expression, (**C**) IGF-1 protein expression, and (**D**) IGF-1 gene expression in STZ-induced DR rats. N: Normal rats with vehicle treatment, DV: Diabetic rats with vehicle treatment, DT: Diabetic rats with TRF treatment, ∆CT: threshold cycle values. Data was parametric and presented as mean ± SD, **p < 0.01, *** *p* < 0.001, *n* = 8

Rats in DV showed 1.54-fold higher retinal VEGF expression compared to N (*p* < 0.001) whereas the rats in DT showed a 1.82-fold lower VEGF protein expression compared to DV (*p* < 0.001) (Fig. 9A). Greater VEGF gene expression was detected in DV compared to N, (2.06-fold, *p* < 0.001). However, lesser expression of VEGF was detected in DT which showed 1.23-fold lower expression compared to DV (*p* < 0.01) (Fig. 9B).

Rats in DV showed 6.01-fold higher retinal IGF-1 expression compared to N (*p* < 0.001) whereas the rats in DT showed a 3.58-fold lower IGF-1 protein expression compared to DV (*p* < 0.01) (Fig. 9C). Greater expression of IGF-1 gene was detected in the retinas of rats from DV compared to those from N (1.62-fold, *p* < 0.01). However, lesser expression of IGF-1 gene was detected in DT which showed a 1.33-fold lower gene expression compared to DV, however the difference did not reach the significant level (Fig. 9D).

#### HIF-1α expression

**Fig. 10:**
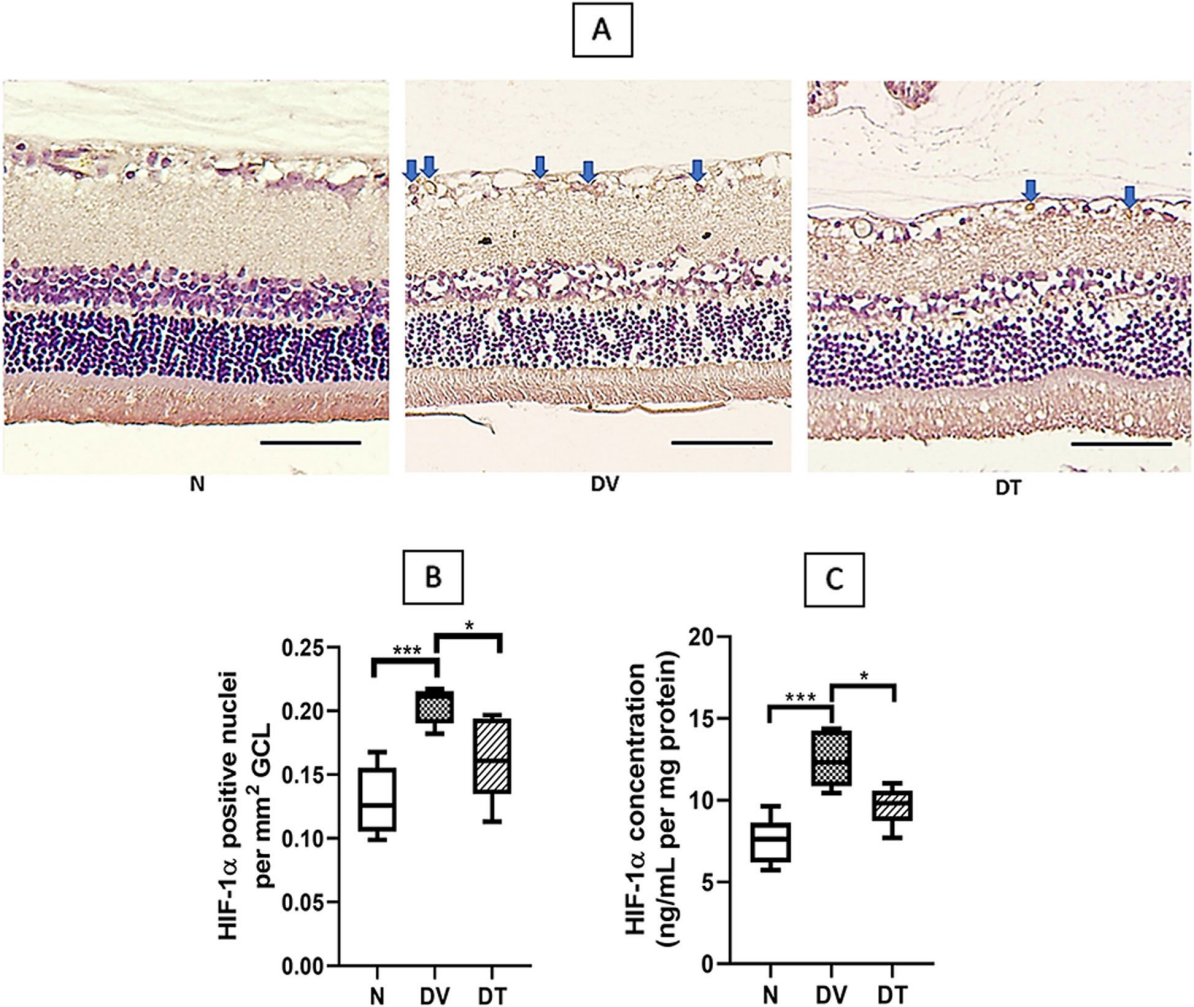
Effect of TRF on retinal HIF-1α expression in STZ-induced DR rats. **A** Representative immunostained retinal sections showing effect of TRF on retinal HIF-1α expression; (**B**) Quantitative expression of the effect of TRF on retinal HIF-1α positive nuclei per mm^2^ (*n* = 4); (**C**) Quantitative expression of the effect of TRF on retinal HIF-1α protein expression measured by ELISA (n = 8). Blue arrows: nuclei positively stained for HIF-1α. N: Normal rats with vehicle treatment, DV: Diabetic rats with vehicle treatment, DT: Diabetic rats with TRF treatment, GCL: Ganglion cell layer. Data was parametric and presented as mean ± SD, *p < 0.05, *** *p* < 0.001 (Scale bar: 50 μm) (Magnification 20X)

Representative microphotographs of immunostained retinal section showing expression of HIF-1α are presented in Fig. 10A.Greater HIF-1α immunostaining was detected in DV compared to N. Quantitatively, there were 1.58-fold higher number of HIF-1α positive nuclei in DV compared to N (*p* < 0.001). Unlike DV, lesser staining for HIF-1α was detected in DT with 1.17-fold lower number of HIF-1α positive nuclei compared to DV (*p* < 0.05) (Fig. 10B).

The expression of HIF-1α protein by ELISA was significantly greater in DV compared to N by 1.34-fold (*p* < 0.001). Whereas lower HIF-1α protein expression was observed in DT compared to DV (1.05-fold, *p* < 0.05) (Fig. 10C).

## Discussion

TRF is a natural compound that is known for its antioxidant, neuroprotective, anti-inflammatory, and anti-angiogenic properties. This study revealed that TRF suppresses retinal expression of the markers of inflammation and angiogenesis in rats with STZ-induced diabetes and this effect of TRF was associated with preservation of retinal morphology and retinal vascular diameter.

As observed in other studies, current study also showed significantly lower weight gain among diabetic rats [57, 58], which could be attributed to negative nitrogen balance due to changes in protein metabolism leading to loss of muscle mass [59]. Relatively greater body weight gain in DT compared to DV correlated with relatively lower blood glucose levels among DT. This effect of TRF in the current study was in line with previous observations [45,6258-]. It is noteworthy that hyperglycemia associated typical changes in retinal vasculature characterizing DR in human such as neovascularization and vitreous hemorrhages are not observed in rats; however, changes in vascular diameter are often noted. Accordingly in the current study, we observed an increase in the diameter of retinal vessels particularly the veins in DV. Similar observations have been made by other researchers using transmission electron microscopy [65], flicker-induced dilation [66] and spectral-domain OCT [67]. However, others have reported contrasting observations such as Miyamoto et al. [68] and Wanek et al. [69], which may be attributed to differences in the strains of animals used, duration of the study, the experimental procedure to induce diabetes and severity of hyperglycemia. Although, we observed an increased vascular diameter, visually evident vascular tortuosity of the vessels was not evident as reported earlier by Gupta et al. [70]. The difference may be due to the shorter duration of diabetes in this study (12 weeks vs. 16 weeks). The retinal vessel diameters in DT were comparable to those in N indicating the efficacy of TRF in attenuating DR-induced retinal vascular changes, which form the basis of progressively increasing morphological and functional impairment.

In order to determine if the effects of TRF on vascular integrity in fact translate into preservation of retinal morphology, we measured the thickness of various retinal layers using H&E-stained section. We observed a reduction in the thickness of all retinal layers in DV and this could be considered as one of the indicators of retinal neurodegeneration involving apoptosis of RGCs, amacrine cells and photoreceptors [71]. Several studies have earlier shown thinning of the retinal layers, affecting the GCL [72, 74], inner layers [73, 75, 76] and outer layers [75, 77] in STZ-induced rat model. Thinning of inner retina is specifically correlated with gradual loss of neural dendrites and synapses [73, 78]. According to other view, prolonged hyperglycemia may induce edematous changes within the retinal layers, which may cause thickening of retinal layers instead of reduction in the thickness [79]. Nevertheless, in the current study, thickness of various retinal layers among diabetic rats treated with TRF was largely comparable to that among normal rats indicating the protective effect of TRF against diabetes induced neurodegeneration.

Inflammation associated with hyperglycemia is one of the key pathophysiological events leading to morphological and functional alterations in DR. Higher levels of inflammatory cytokines such as IL-1β, IL-6, MCP-1 and TNF-α have been observed in the ocular fluids of DR patients compared to diabetic patients without DR [80]. Moreover, the increase in the expression of inflammatory cytokines and chemokines tends to correlate positively with the progression in the severity of DR [81]. In accordance with these observations, we observed an increased retinal gene and protein expressions of IL-1β, IL-6, TNF-α, IFN-γ, MCP-1, and iNOS in DV compared to N, while the same were suppressed in DT indicating the anti-inflammatory effects of TRF.

Role of various cytokines in DR retinopathy has been widely studied. IL-1β, one of the most prominent inflammatory cytokines in DR, promotes the expression of chemokines and macrophage recruitment, thereby facilitating the degenerative changes found in DR [82]. TNF-α plays an important role in the early and late stage of blood-retinal barrier breakdown in DR by increasing the mitophagy-associated cell apoptosis [83, 84]. In a recent study, TNF-α, alongside with IFN-γ, was reported to play a role in the degradation of platelet endothelial cell adhesion molecule-1 (PECAM-1) in DR [85]. PECAM-1 is a marker for maintenance of vascular integrity [86], and its degradation is associated with cell apoptosis in DR [87]. TNF-α activation may also be influenced by the increased production of IFN-γ [88]. IFN-γ induces M1 macrophage polarization [89] by elevating IFN-regulatory factors (IRFs) production [90], and it activates NFκB and STAT-1 [91], which mediate the upregulation of inflammatory cytokines, for instance, IL-1β, IL-6, and TNF-α [92]. IFN-γ also independently stimulates VEGF expression in retinal pigment epithelial cells via the PI-3 K/Akt/mTOR/p70 S6 kinase pathway [93]. Polymorphism in the intron of the IFN-γ gene that leads to increased IFN-γ expression was reported to be highly associated with proliferative DR in type 2 DM patients [94]. In the current study, although it is likely that TRF could have directly suppressed the expression of various inflammatory cytokines, it may partly be also secondary to lower expression of retinal IFN-γ [95]. Among the other cytokines measured in the current study, MCP-1 contributes to retinal inflammation in DR through recruitment and activation of monocytes and macrophages [96]. Reduced MCP-1 expression in response to TRF treatment observed in this study was in accordance with previously observed effects of TRF on adipocytes [64, 97], keratinocytes [98], colon cancer cells [99] and liver cells [100]. IL-6 produces immunomodulatory effects via JAK-STAT pathways by stimulating trans-signaling and contributes to improved glucose uptake and metabolism [101]. It is likely that slight improvement in blood glucose level and body weight gain in TRF treated group is related to increased IL-6 expression. Similar observation was not made in DV despite high IL-6 expression, perhaps because of significant upregulation of other counterproductive cytokines which was not the case in DT. In this regard it is also notable that reduced expression of iNOS and other cytokines in DT is likely to translate into reduced retinal oxidative stress as shown earlier by Kuhad and Chopra [31] and Abdul Nasir et al. [45], which in turn protects against hyperglycemia associated vascular and morphological alterations.

Reduced expression of cytokines among TRF treated rats in the current study was associated with reduced NFκB activation, a transcription factor known to promote expression of pro-inflammatory proteins. Hence it is likely that the reduced expression of various cytokines by TRF, at least partially, was secondary to its effects on NFκB. In fact, TRF is known to modulate NFκB signaling pathways through several mechanisms [102], one of which involves upregulation of PPARα and PPARγ expression [103]. PPARα and PPARγ inhibit the activation of NFκB through increased expression of IκBα, PTEN, and increased antioxidants activity [104, 105]. Matsunaga et al. [97] observed that γ-tocotrienol upregulated the PPARγ expression that resulted in the inhibition of NFκB activation in TNFα-treated adipocytes. Shen et al. [106] also reported the upregulation of PPARγ and PPARα by δ- tocotrienol in LPS-induced macrophages. Tocotrienol may also suppress NFκB activation by upregulating the A20 molecule and cylindromatosis (CYLD) gene, which are the negative regulator of NFκB activation [107].

Retinal inflammation in DR is associated with increased expression of the markers of angiogenesis. In fact, Niu et al. [108] observed that MCP-1 induced MCPIP (MCP-1-induced protein) transcription factor in human peripheral blood monocytes, which upregulated the downstream genes responsible in the angiogenic process. MCP-1 also promotes VEGF expression through activation of the CCR2/ILK/MEK1/2 signaling pathway and downregulation of miR-29c, a tumor suppressor microRNA (Lien et al. [109]. Reduction of inflammation by TRF, as seen in this study, may be one of the reasons for the reduction in VEGF gene and protein expression. At post-transcriptional level, reduction of VEGF levels by tocotrienols was associated with suppression of Ras-Raf-MEK-ERK signaling pathway [110]. It is known that tocotrienols activate the p53 signaling pathway in several cancer models [111, 112]. p53 suppresses the IGF-1/Akt pathway signaling [113]. IGF binding protein-3 (IGF-BP3) acts by binding to the free IGF-1, which prevents IGF-1 binding to its receptor and therefore inhibits the activation of the downstream signaling pathway [114]. Hence, in this study, tocotrienols may have downregulated IGF-1 expression through activation of p53. HIF-1α, which acts as modulator of pro-angiogenic factors including VEGF [115], was also reduced in TRF-treated group compared to the DV. These findings of the current study are in agreement with previous studies showing that tocotrienols suppress HIF-1α activation in the experimental models of prostate [116] and colorectal carcinoma [37].

## Conclusions

This study showed that 12 weeks of oral treatment of TRF in rats with STZ-induced diabetes reduces expression of the markers of retinal inflammation including IL-1β, IL-6, TNF-α, IFN-γ, MCP-1, and iNOS. It also suppresses expression of VEGF, IGF-1 and HIF-1α that play a role in hyperglycemia associated angiogenesis. Suppression of the activation of NFκB signaling may at least partially underlie the effects of TRF on the expression of the markers of inflammation and angiogenesis. Importantly, these effects of TRF were reflected in the preservation of retinal vascular diameters and morphology. Overall, TRF provided protection against retinal inflammation and angiogenesis in rats with STZ-induced diabetes. These effects of TRF were associated with protection against diabetes-induced changes in retinal venous diameter and retinal morphology.

## Data Availability

The datasets supporting the conclusions of this article are available upon request to the corresponding author.
